# The Paper-Toss Test: enhancing bedside recognition of corticobasal syndrome

**DOI:** 10.3389/fneur.2025.1634177

**Published:** 2025-12-08

**Authors:** Gero Lueg, Thomas Duning, Markus A. Hobert, Alexander Rösler, Sara Peranovic, Rainer Wirth, Julia Krämer

**Affiliations:** 1 Department of Geriatric Medicine, Marien Hospital Herne, Ruhr University Bochum, Herne, Germany; 2 Department of Neurology, Klinikum Bremen-Ost, Bremen, Germany; 3 Department of Neurology, University Medical Center Schleswig-Holstein, Campus Kiel and Kiel University, Kiel, Germany; 4 Department of Neurology, University Medical Center Schleswig-Holstein, Campus Lübeck and University of Lübeck, Lübeck, Germany; 5 Department of Geriatric Medicine, Bethesda Krankenhaus Hamburg Bergedorf, Hamburg, Germany; 6 Department of Neurology, University Hospital Münster, Münster, Germany

**Keywords:** corticobasal syndrome, CBS, PSP, CBD, apraxia

## Abstract

**Introduction:**

The diagnosis of corticobasal syndrome (CBS) is challenging due to its clinical overlap with other neurodegenerative disorders. Ideomotor apraxia is a core feature of CBS and often presents asymmetrically, but is often under-recognized in the clinical setting.

**Methods:**

The “Paper-Toss Test” (PTT) is administered to a video-based case series of four patients with different CBS pathologies (Alzheimer’s disease, progressive supranuclear palsy, and corticobasal degeneration) and involves instructions to throw a paper ball with each hand. A positive test result is indicated by the presence of specific performance deficits of the patient’s affected side such as an absent or delayed release of the paper ball or an interrupted throwing motion.

**Results:**

All patients exhibited a positive PTT, independent of the underlying cause of CBS.

**Discussion:**

This study proposes the PTT (Paper-Toss Test) as a bedside tool for the detection of unilateral ideomotor apraxia, facilitating the diagnosis of CBS. These deficits correspond to the known impairments in transitive gestures and disturbances of the neuronal apraxia network in CBS patients. These results support further investigation and validation of the PTT in clinical practice.

## Introduction

The clinical diagnosis of corticobasal syndrome (CBS) is challenging due to its complex presentation with diverse clinical and pathological features (1). The prevalence of CBS overlaps with neurodegenerative diseases such as corticobasal degeneration (CBD), progressive supranuclear palsy (PSP), Alzheimer’s disease (AD), frontotemporal lobar degeneration (FTLD), and Creutzfeldt-Jakob disease (CJD) (1).

According to a recent study, the diagnosis of CBD and PSP is delayed by up to 4 years compared to Parkinson’s disease because of late referral to movement disorders and dementia specialists (2). In this study, we therefore propose a test that has been utilized in our clinical practice for many years and that can be employed to facilitate the diagnosis of CBS (3). It is important to note that the “Paper-Toss Test” (PTT) is not intended to replace a comprehensive apraxia assessment, but rather to trigger further diagnostic testing. The four video-based cases presented herein include two patients with CBS/CBD, one patient with CBS/AD, and one patient with CBS/PSP. The subsequent videos illustrate the examination of the patients and the application of the PTT.

## Methods

All four female patients were referred to the Department of Geriatric Medicine at the Marienhospital Herne, Ruhr University Bochum, and the Department of Neurology at the University Hospital of Münster. They were admitted to the clinic for further examination and treatment after receiving a diagnosis of PD, with no prior diagnosis of CBS from their primary care physicians. All patients were right-handed. The diagnosis of different clinical phenotypes associated with the pathology of CBS was made in accordance with established diagnostic criteria (see Table 1) (4).

**Table 1:** A summary of the patients’ baseline characteristics and clinical findings.

Variable	Case 1	Case 2	Case 3	Case 4
Age (y)	78	76	67	65
Symptom duration at diagnosis (y)	2	1	3	1
Clinical phenotype; Diagnosis	Probable CBS; CBS/AD	Probable CBS; CBS/CBD	Probable CBS; CBS/CBD	PSP-Syndrome/ CBS/PSP
Clinical features	Verbal memory impairment, reduced verbal fluency. Asymmetric presentation of limb rigidity, dystonia, limb apraxia and alien limb of the right arm, mild gait ataxia	Asymmetric presentation of limb rigidity, limb dystonia, limb apraxia and alien limb of the right arm, mild gait ataxia	Asymmetric presentation of limb rigidity and dystonia of the left arm and leg, limb apraxia, alien limb phenomenon of the left arm, severe gait ataxia	Asymmetric hypokinetic-rigid syndrome, dystonia and alien limb of the left arm and leg, vertical gaze palsy, gait ataxia, dysphagia, impaired verbal memory
Levodopa response	No to 800 mg/d	No to 800 mg/d	No to 1,200 mg/d	No to 500 mg/d
Family history of movement disorder	No	No	No	
CSF beta-amyloid 1–42 (>599 pg/mL)	400 pg/mL	910 pg/mL	658 pg/mL	1,328 pg/mL
CSF Total tau protein (<500 pg/mL)	710 pg/mL	450 pg/mL	168 pg/mL	275 pg/mL
CSF Phospho-Tau (<57 pg/mL)	45	33	NA	NA
MoCA	14/30	22/30	NA	NA
MMST	NA	NA	26/30	NA
IAT (< 27/30 points indicates ideomotor apraxia for finger and hand postures)	12/30	15/30	NA	25/30
AST (< 9/12 points indicates apraxia for meaningless, communicative and tool-related gestures)	Right hand 5/12, Left hand 10/12	Right hand 4/12, Left hand 9/12	NA	NA
CAS	NA	NA	49/80	NA
CAS 1.1: Imitation of meaningless individual movements			10/20	
CAS 1.2: Imitation of meaningless sequences of movements			12/20	
CAS 2.1: Imitation of meaningful gestures			19/20	
CAS 2.2: Pantomime of object use			18/20	
Imaging findings (MRI)	Bilateral hippocampal atrophy, asymmetric cortical atrophy of the left superior parietal lobe	Asymmetric cortical atrophy of the left superior parietal lobe, atrophy of the corpus callosum	Asymmetric cortical atrophy of the right superior parietal lobe, atrophy of the corpus callosum	Generally extended cerebrospinal fluid spaces without typical signs of atrophy
Imaging findings (FDG-PET)	NA	NA	NA	Bifrontal, including premotor and precentral cortex, and midbrain hypometabolism

CSF, Cerebrospinal Fluid; MoCA, Montreal Cognitive Assessment; MMSE, Mini-Mental State Examination; IAT, Ideomotor Apraxia Test; AST, Apraxia Screening of Tulia; CAS, Cologne Apraxia Screening; MRI, Magnetic Resonance Imaging; FDG-PET, Fluorodeoxyglucose Positron Emission Tomography; NA, not available.

In the PTT, the examiner instructs the patient to throw a balled-up piece of paper in a high arc. It is recommended that the initial attempt should be carried out with the side not affected by CBS. While mirror movements may emerge on the nonaffected side, the paper toss should be performed with ease. In the second attempt, the patient is asked to take the ball with the side affected by CBS and to throw it. Compared to the first trial, the patient experiences often noticeable difficulty in taking the paper ball from the examiner. For example, the unaffected side is used to facilitate this process. The test is considered pathological (positive) if the following errors occur during the test:

1. The patient is unable to release the paper ball at will.
2. The release of the paper ball is initiated with a notable time delay in comparison to the opposite side, or it drops to the ground due to gravity.
3. The throwing attempt is stopped mid-air or the limb remains in a dystonic posture.

## Results

### Case 1

The patient exhibited impaired verbal memory and slowed speech for 2 years, along with involuntary levitation of the right arm along with a sense of alienation of the right hand in the last 6 months. Analysis of the cerebrospinal fluid (CSF) showed signs of AD. Further results are shown in Table 1. Supplementary Video 1, Case 1, Segment 1, presents a dystonic posture of the right arm and gait disturbances. Segment 2 illustrates the bedside apraxia test using a variation of the Goldenberg hand posture and finger configuration imitation tests (5). Imitation of meaningless gestures was significantly impaired for both finger and hand gestures. In the PTT (Segment 3), the patient exhibited mirror movements when grasping the paper ball; however, the throwing movement was executed successfully. When the patient attempted to throw the paper ball with the affected right side, pronounced mirror movements recurred, and the paper ball could not be released at will due to finger apraxia.

### Case 2

The patient was admitted to our hospital after a fall that resulted in a left rib contusion. The patient reported a gradual decline in gait stability and difficulties using her right arm for approximately 1 year, thereby necessitating an increase in use of her left hand for daily tasks (see Table 1). During imitation tasks in Supplementary Video 1, Case 2, Segment 1, the patient shows dystonic posturing of the right arm. Imitation of meaningless gestures is displayed in Segment 2. The patient is incapable of positioning her hands and fingers appropriately, despite visual and motor attempts to correct her finger position. In Supplementary Video 1, Case 2, Segment 3, the paper toss test is performed. On the affected right side, the throwing attempt is significantly impaired compared to the unaffected side. The PTT demonstrates that the paper ball is more likely to be dropped than thrown with precision.

### Case 3

The patient presented to our hospital for adjustment of dopaminergic therapy after being diagnosed with PD 3 years earlier. During the clinical examination the patient reported a sense of alienation of the left arm and leg with reduced voluntary motor control. Over the past year, she has lost her gait stability. Supplementary Video 2, case 3, segment 1, demonstrates the loss of gait stability and a dystonic posture of the left arm. The patient is then instructed to shake hands with the examiner using only one hand. During this attempt, co-innervation and pronounced mirror movements are observed. Furthermore, pronounced positioning disturbances become apparent during the imitation of hand and finger gestures. Supplementary Video 2, case 3, segment 2 shows the Paper-Toss Test. When the patient attempts to grasp the paper using the affected side, she first places the paper ball in the unaffected hand and exhibits pronounced mirror movements. Subsequently, the paper ball could not be released at will and is more likely to be dropped accidentally with the affected side.

### Case 4

The patient presented to our hospital with a progressive memory impairment, behavioral changes and loss of the ability to walk and falls for 1 year. A clinical evaluation revealed an asymmetric hypokinetic-rigid syndrome, a dystonic posture with bradykinesia and an alien limb of the left arm and leg, a vertical gaze palsy, and gait ataxia. To confirm the diagnosis, an FDG-PET was performed, which revealed the characteristic pattern of a PSP/CBD syndrome (see Table 1). Supplementary Video 2, Case 4, Segment 1, reveals dystonic posturing of the left arm and bradykinesia of the left leg. In Segment 2, the paper toss is performed flawlessly on the non-affected right side. On the affected side the ball throw is disrupted mid-movement and the ball is not thrown with ease, but is rather pushed away.

## Discussion

Apraxia is a clinical hallmark of CBS. The proposed PTT is a diagnostic instrument employed to test for the presence of asymmetrical ideomotor apraxia. The test involves the subjects throwing a piece of paper on command, which is a transitive gesture that involves tool use and requires intact visuomotor transformation. The outcome of the test primarily depends on intact praxis of the hand and fingers.

In the present cases, the PTT consistently elicited characteristic errors on the affected side, including delayed or absent release of the paper ball, accidental dropping due to loss of distal control, dystonic halts or interruptions of the throwing movement, and abnormal distal as well as proximal limb postures with disrupted trajectories. These error profiles correspond to the qualitative descriptions of praxis deficits in CBS/CBD, where distal (limb-kinetic) impairments frequently predominate over proximal control (1, 6–9).

Notably, our CBD-CBS cases showed prominent distal release failures and dropping, whereas the PSP-CBS case mainly displayed dystonic halts and interrupted trajectories rather than marked distal release deficits. This clinical pattern is in line with comparative studies reporting more severe distal praxis deficits in CBS/CBD and less pronounced distal–proximal gradients with predominant ideomotor/sequence errors in PSP (7, 10). Imaging data support this dissociation (11, 12) CBS typically shows asymmetric frontoparietal and callosal involvement, while PSP emphasizes midbrain and superior cerebellar peduncles within a shared premotor–callosal network.

These anatomical patterns can be further interpreted within the three-stream model of praxis (13). In this framework, the dorso-dorsal stream supports trajectory planning and body-schema integration, the ventro-dorsal stream mediates object-related hand shaping and release, and the ventral stream subserves semantic and communicative aspects of gesture. The error profiles observed in the PTT map well onto this organization: delayed or absent release and dropping of the ball reflect ventro-dorsal dysfunction (inferior parietal and ventral premotor regions), while abnormal proximal postures and disrupted throwing trajectories indicate dorso-dorsal impairment (superior parietal and dorsal premotor regions). The ventral stream plays a less prominent role in this task, consistent with the transitive, object-directed nature of the PTT.

All patients also exhibited ideomotor apraxia on standardized testing, a feature that is well recognized in CBS but not disease-specific, as it is also common in AD and other dementias (14). By contrast, the PTT elicited additional, side-specific distal kinetic errors (delayed or absent release, dropping), which are not typically emphasized in AD. This distinction underlines the added diagnostic value of the PTT as a bedside tool, complementing established apraxia batteries.

In our experience, we have not observed a positive PTT in patients without CBS. The positive result of the PTT is therefore not solely due to the presence of limb apraxia or movement disorder, as CBS is a complex syndrome that is associated with complex sensorimotor dysfunction in addition to apraxia (15). To address this gap in knowledge, a prospective structured clinical survey is being planned. This survey will include standardized apraxia testing and a comparison of patients with positive and negative PTT in atypical Parkinson’s syndromes and movement disorders.

The study is not without limitations; it is retrospective and has yet to be correlated with established apraxia tests. To validate the findings, it is necessary to use a battery of apraxia tests with imitation of hand and finger movements, transitive and intransitive gestures, and proximal and distal movements, separately for the affected and unaffected side of CBS.

The PTT is a straightforward clinical tool with the potential to enhance diagnostic awareness and prompt further diagnostic tests for CBS, a particularly salient feature in cases of CBD and PSP.

## Data Availability

The raw data supporting the conclusions of this article will be made available by the authors, without undue reservation.

## Supplementary material

SUPPLEMENTARY VIDEO 1 Case 1/ Segment 1: Dystonic posture of the right arm and gait ataxia. Segment 2: Imitation of hand- and finger postures. Segment 3: Positive Paper-Toss Test (PTT) with inability to release the paper ball at will. Case 2/ Segment 1: Dystonic posture of the right arm. Segment 2: Insufficient imitation of meaningless gestures. Segment 3: Positive PTT with insufficient throwing attempt.

SUPPLEMENTARY VIDEO 2 Case 3 / Segment 1: Bedside apraxia testing with performance of a handshake and meaningless gestures. Segment 2: Positive PTT (Paper-Toss Test) where the paper ball could not be released at will. Case 4 / Segment 1: Dystonic posture with bradykinesia of the left arm and leg. Segment 2: Positive PTT (Paper-Toss Test) with disrupted throw attempt mid-movement.
